# Long- and short-term effects of fecal microbiota transplantation on antibiotic resistance genes: results from a randomized placebo-controlled trial

**DOI:** 10.1080/19490976.2024.2327442

**Published:** 2024-03-13

**Authors:** Armin Rashidi, Maryam Ebadi, Tauseef Ur Rehman, Heba Elhusseini, David Kazadi, Hossam Halaweish, Mohammad H. Khan, Andrea Hoeschen, Qing Cao, Xianghua Luo, Amanda J. Kabage, Sharon Lopez, Shernan G. Holtan, Daniel J. Weisdorf, Chang Liu, Satoshi Ishii, Alexander Khoruts, Christopher Staley

**Affiliations:** a Clinical Research Division, Fred Hutchinson Cancer Center and Division of Oncology, University of Washington, Seattle, WA, USA; b Division of Hematology, Oncology, and Transplantation, Department of Medicine, University of Minnesota, Minneapolis, MN, USA; c Department of Radiation Oncology, University of Washington and Fred Hutchinson Cancer Center, Seattle, WA, USA; d Department of Medicine, University of Minnesota, Minneapolis, MN, USA; e Department of Surgery, University of Minnesota, Minneapolis, MN, USA; f Biostatistics Core, Masonic Cancer Center, University of Minnesota, Minneapolis, MN, USA; g Division of Biostatistics, School of Public Health, University of Minnesota, Minneapolis, MN, USA; h Division of Gastroenterology, Hepatology, and Nutrition, Department of Medicine, University of Minnesota, Minneapolis, MN, USA; i Department of Soil, Water, and Climate, BioTechnology Institute, University of Minnesota, MN, USA; j Biotechnology Institute, University of Minnesota, Minneapolis, MN, USA; k Center for Immunology, University of Minnesota, Minneapolis, MN, USA

**Keywords:** Acute myeloid leukemia, antibiotic resistance gene, fecal microbiota transplantation, hematopoietic cell transplantation

## Abstract

In small series, third-party fecal microbiota transplantation (FMT) has been successful in decolonizing the gut from clinically relevant antibiotic resistance genes (ARGs). Less is known about the short- and long-term effects of FMT on larger panels of ARGs. We analyzed 226 pre- and post-treatment stool samples from a randomized placebo-controlled trial of FMT in 100 patients undergoing allogeneic hematopoietic cell transplantation or receiving anti-leukemia induction chemotherapy for 47 ARGs. These patients have heavy antibiotic exposure and a high incidence of colonization with multidrug-resistant organisms. Samples from each patient spanned a period of up to 9 months, allowing us to describe both short- and long-term effects of FMT on ARGs, while the randomized design allowed us to distinguish between spontaneous changes vs. FMT effect. We find an overall bimodal pattern. In the first phase (days to weeks after FMT), low-level transfer of ARGs largely associated with commensal healthy donor microbiota occurs. This phase is followed by long-term resistance to new ARGs as stable communities with colonization resistance are formed after FMT. The clinical implications of these findings are likely context-dependent and require further research. In the setting of cancer and intensive therapy, long-term ARG decolonization could translate into fewer downstream infections.

## Introduction

Intestinal colonization with multidrug-resistant organisms (MDROs) in patients undergoing allogeneic hematopoietic cell transplantation (alloHCT) or receiving anti-leukemia induction chemotherapy has been associated with an increased risk of systemic infections and mortality.^1–4^ Antibiotic exposure seems to be a major driver of MDRO colonization.^4^ One potential approach to decolonize the gut from MDROs is fecal microbiota transplantation (FMT), via which intestinal microbial communities from a healthy donor are transferred to the patient. The efficacy of FMT in treating recurrent *Clostridiodes difficile* infections (rCDI) has suggested its potential to reduce MDRO colonization in alloHCT recipients and AML patients.

Thus far, the effects of FMT on the MDRO carriage in patients with acute leukemia or those undergoing alloHCT have been examined in only a few small series.^5–8^ While a few specific MDROs with known clinical significance were evaluated in most previous studies, large-scale data using expanded panels of antibiotic-resistance genes (ARGs) are unavailable. In addition, short- and long-term changes in ARGs after FMT are largely unknown. The decline in antibiotic pressure over time after the initial phase of HCT or induction chemotherapy may influence ARG dynamics. Distinguishing these changes from FMT effects is best done in the context of a randomized placebo-controlled trial. This has not been performed yet.^9^

In the current study, we took advantage of longitudinal pre- and post-treatment stool samples in our recent randomized double-blind trial of oral, standardized, encapsulated, third-party FMT product vs. placebo in two independent cohorts of patients with AML undergoing induction chemotherapy and allogeneic HCT recipients. The treatment was administered at the time of neutrophil engraftment. A total of 100 patients received study treatment. FMT was safe and ameliorated intestinal dysbiosis.^10^ The large number of ARGs analyzed (47 ARGs), access to longitudinal stool samples collected over 9 months, and the randomized design of the trial allowed us to address the three knowledge gaps of interest.

## Methods

### Trial design summary and previously reported key findings

The trial protocol (ClinicalTrials.gov identifier: NCT03678493) and informed consent were approved by the University of Minnesota Institutional Review Board and complied with local regulations and the Declaration of Helsinki. Trial protocol, design, eligibility criteria, and procedures for this trial were previously published.^10^ Briefly, adults undergoing inpatient, T-replete allogeneic HCT for any indication (HCT cohort) or inpatient chemotherapy for AML (AML cohort) underwent non-stratified double-blinded randomization in a 2:1 ratio to receive third-party FMT or placebo in the form of 5 oral capsules taken at once after neutrophil recovery and at least 2 days after discontinuation of antibacterial antibiotics. Each patient received material (5 capsules) manufactured using Good Manufacturing Practice protocols from one of the four donors, as previously described.^11^ The protocol excluded donor stool that contained detectable vancomycin-resistant *Enterococcus*, extended-spectrum beta-lactamase expressing *Enterobacteriaceae*, carbapenem-resistant *Enterobacterales*, and methicillin-resistant *Staphylococcus aureus*. The preprocessing weight of donor stool was 50 g. Each FMT capsule contained ≥ 1 × 10^11^ bacteria with ≥ 40% viability. We chose this dose based on our prior experience with patients with rCDI. A dose as low as ~ 2 × 10^11^ (2 capsules) was effective in treating rCDI in our first trial.^11^ The dose of ~ 5 × 10^11 bacteria (5 capsules) is currently the standard dose in our CDI clinical practice.^12^ Patients were followed for 9 months. The primary endpoint was all-cause infection rate within 4 months. We enrolled 74 HCT patients (FMT, 49; placebo, 25) and 26 AML patients (FMT, 18; placebo, 8). Stool samples were collected in 95% ethanol at baseline (before starting conditioning; T0), before the first dose of FMT/placebo (T1), 10 days after the first dose of FMT/placebo (T2), 28 days after the first dose of FMT/placebo (T3), and at 9 months (T4). If antibacterial antibiotic exposure occurred after dose 1 but before the T2 or T3 timepoint, the sample was not collected and dose 2 was administered. The trial did not reach its primary endpoint; the numerical decrease in infection rate in the FMT arm did not reach statistical significance.^10^

## ARG assay

After 16S rRNA gene sequencing for the preplanned endpoints of the trial, 226 samples with remaining DNA were used for microfluidic quantitative PCR (MFQPCR) to simultaneously detect and quantify 47 ARGs including those for beta-lactams, quinolones, vancomycin, and carbapenem resistance.^13^ The ARG panel is provided in Table 1. This panel includes 16 classes of ARGs based on their function and antibiotic groups to which they confer resistance. Seven ARGs (*ermB*, *qnrA*, *qnrB*, *catB8*, *floR*, *tetL*, and *tetM*) and specific subtypes of 4 ARGs (*blaVIM*, *blaKPC*, *blaOXA*, and *blaSHV*) on the panel are considered current threats to human health^14^ (also see https://www.cdc.gov/drugresistance/biggest-threats.html). These genes are shown in bold thereafter. BioMark HD System (Fluidigm) and 96.96 Dynamic Array IFCs (Fluidigm) were used for analysis. Serial dilutions (2 × 10° to 2 × 10^6^ copies/μL) of the mixture of synthetic DNA fragments containing each target gene sequence were included in the analysis to generate standard curves. Specific target amplification (STA) was done with 14 PCR cycles to increase the target DNA molecule prior to MFQPCR.^15^ Sample DNA, gBlock standard mixtures, and no template controls were subjected to STA. Quantitative results obtained by MFQPCR were analyzed using Real-Time PCR Analysis (Fluidigm) software version 4.1.2.^15^ All samples were run in duplicate, and their average gene content for each ARG (gene copies/μL DNA) was calculated using standard curves of the respective genes and then logarithmically transformed. Gene copy numbers and related clinical metadata are provided in **supplementary data S1**.

**Table 1.** The ARG panel and resistance spectrum.

Class	ARGs
Sulfonamide resistance	sul1, sul2, sul3
Macrolide resistance	ereB, **ermB**, ermF, mefE
Aminoglycoside resistance	aacA, aadD, aadA5
Quinolone resistance	**qnrA**, **qnrB**
Chloramphenicol resistance	**catB8**, cmlB, **floR**
Vancomycin resistance	vanA, vanB
Carbapenem resistance	blaVIM*, blaKPC*, blaNDM-1, blaOXA*, IMP-13
Non-carbapenem beta-lactam resistance	ampC, blaSHV*, CTX-M32, blaNPC
Rifampicin resistance	arr2
Tetracycline resistance	tetA, **tetL**, **tetM**, tetS, tetW, tetX
Streptomycin resistance	strB
Trimethoprim resistance	dfr13
Heavy metal resistance	cadA, chrA, copA, merA, nikA, rcnA
Integrons (increasing ARG spread)	intl1, intl2, intl3
Quaternary ammonium compound resistance (also increasing ARG spread)	qacF
Multiple antibiotic resistance through efflux pump	acrD, mexB

ARG genes shown in bold are considered current threats to human health (Rank I). Specific subtypes of ARGs marked with * are Rank I.

## Statistical analysis

The two arms (FMT vs. placebo) were compared at each timepoint. Both categorical (presence/absence; chi-squared test with a Fisher’s exact test when appropriate) and quantitative (gene copy number; Wilcoxon rank sum test for independent samples) analyses were performed on a per-ARG basis. In each categorical comparison, ARGs with levels below the quantification threshold in > 25% of the samples were omitted as long as their frequency of absence between the two groups was not statistically different (*p* < 0.05).

In quantitative analysis, ARGs with levels below the quantification threshold in > 50% of the samples were omitted, again as long as their frequency of absence between the two groups was not statistically different (*p* < 0.05). Then, the half-minimum imputation method was used for zero-replacement for non-omitted ARGs. All values were normalized for 16S rRNA gene copies/μL DNA from quantitative PCR. To evaluate the dynamics of each ARG between the pretreatment timepoint (T1) and short- (T2, T3) and long-term (T4) post-treatment timepoints, a mixed-effects linear regression model was built. The dependent variable was the normalized ARG level. Fixed effects were timepoint (T2, T3, or T4 compared to T1), treatment arm (Placebo vs. FMT), and an interaction between these two terms. Patient number was the random effect. If an interaction effect was not suggested, the interaction term was removed and the effects of treatment arm and timepoint were estimated. However, if an interaction term appeared to be present in the mixed model, the respective ARG was examined further, once in the FMT arm and once in the placebo arm, using a simplified mixed model with ARG level as the dependent variable, timepoint as the sole fixed effect, and patient number as the random effect.

In the analyses requiring multiple testing, *p* values were corrected and transformed to q values using the Benjamini-Hochberg method.^16^ q < 0.10 was considered statistically significant. R 4.2.0 was used for all analyses. The *nlme* package in R was used for mixed modeling, and the MLE-based inference method to derive *p* values and 95% confidence intervals.

## Results

**Figure 1.**
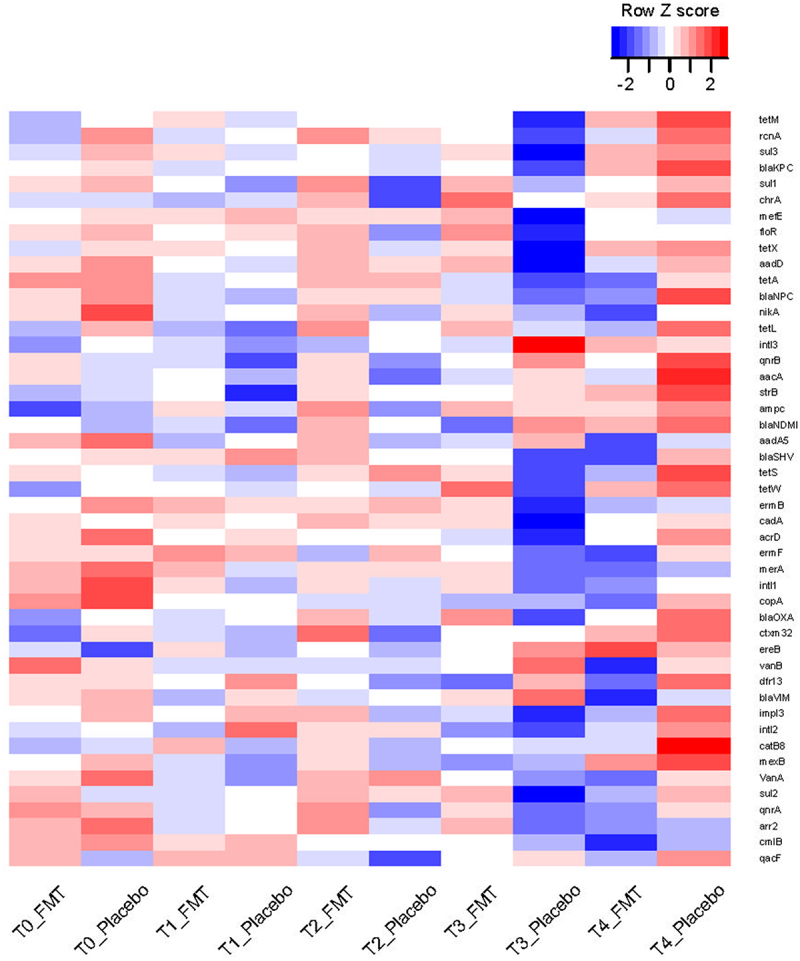
Heatmap of ARGs over time.

A total of 224 samples were included in the analysis, 174 from the HCT cohort and 50 from the AML cohort. Of these, 159 samples were from the FMT arm and 65 from the placebo arm. Temporal distribution of these samples was 57 samples at T0, 49 samples at T1, 50 samples at T2, 27 samples at T3, and 41 samples at T4. Two T4 samples were collected from patients who received more than 1 dose of FMT. Because of the intervening doses of FMT between dose 1 and the T4 sample, these two samples were not included. From each patient at each timepoint, either 0 or 1 sample was available for analysis, with a median (mean) of 3 samples per patient from the possible 5 timepoints. In addition to the lack of sufficient DNA for analysis, other reasons for the lack of samples at the other timepoints included patient refusal to provide a sample, logistic challenges for patients followed primarily by the referring oncologist (sometimes from other states), and specific restrictions enforced by our institution during the COVID-19 pandemic. In the final analyzed list, all but 5 patients received only 1 dose of FMT or placebo. Four of these patients were in the placebo arm and only 1 was in the FMT arm. These patients did not have a T4 sample, thus their post-dose antibiotic exposures did not contribute to our results. Antibiotic exposures before dose 1 (between T0 and T1) were reported in our original report and were comparable between the two treatment arms.^10^ The prevalence of each ARG among samples at each timepoint in each arm is visualized in Figure 1.

Each row shows an ARG and each column a timepoint associated with a treatment arm. The color gradient indicates the frequency of each ARG in samples collected at each timepoint in each arm, after scaling row-wise. More intense red colors indicate higher frequencies while more intense blue colors indicate lower frequencies. The treatment arms are FMT vs. placebo. T0: baseline, T1: pre-treatment, T2: 10 days after treatment, T3: 28 days after treatment, T4: long-term (9 months)

**Figure 2.**
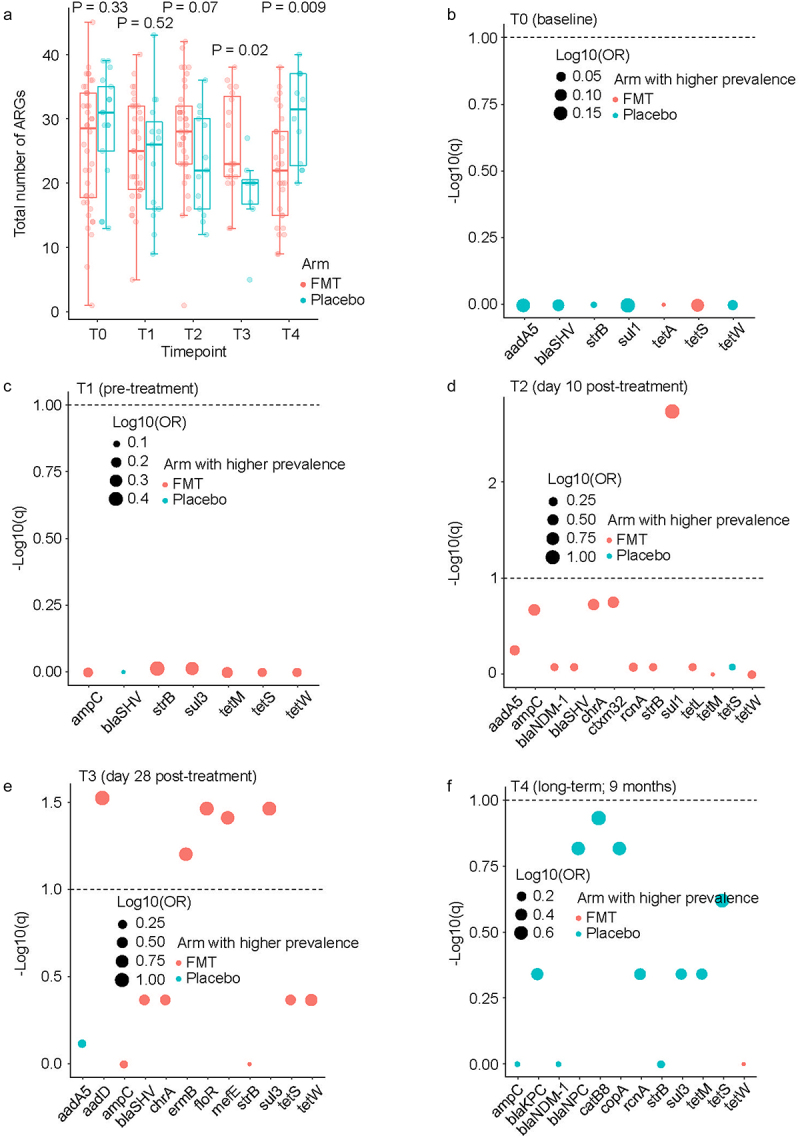
Comparison between the two arms for prevalence of each ARG at each timepoint.

Comparing the total number of ARGs between the two arms at each timepoint using a Wicoxon rank sum test (Figure 2a), no differences were found between FMT and placebo arms at T0 (p = 0.33) or T1 (p = 0.52). At T2, there was a trend for a larger number of ARGs in the FMT arm (FMT: median 28 genes, range 1–42; placebo: median 22 genes, range 12–36; p = 0.07). This trend became significant at T3 (FMT: median 23 genes, range 13–38; placebo: 20 genes, range 5–27; p = 0.02) and was reversed at T4 (FMT: median 22 genes, range 9–38; placebo: 32 genes, range 20–40; p = 0.009). To quantify the magnitude of difference in the prevalence of each ARG between the two arms at each timepoint, we used a chi-squared test, followed by correction of *p* values for multiple testing (Figure 2b-f). No significant differences were found between the two arms at T0 or T1. This was expected from the randomized design of the trial and the fact that T0 and T1 timepoints preceded the study intervention. At T2, the only significant ARG was *sul1*, with a higher prevalence after FMT vs. placebo (89% vs. 31%, respectively). At T3, 5 ARGs were significantly more prevalent after FMT vs. placebo: *aadD* (79% vs. 13%), *ermB* (48% vs. 0), *floR* (74% vs. 13%), *mefE* (68% vs. 13%), and *sul3* (84% vs. 25%). Finally, at T4, no ARG was significantly different between the two arms. There was no significant difference between the two arms for the proportion of the 11 high-risk ARGs (listed in Methods under “ARG assay”) relative to all ARGs present at each timepoint (supplementary Fig. S1).

Panel a compares the total number of ARGs between the two arms at each timepoint, using a Wilcoxon test. The horizontal line in each boxplot shows the median. Panels b-f quantify the magnitude of difference in the prevalence of each ARG between the two arms at each timepoint, a chi-squared test was used, followed by false discovery correction of *p* values for multiple testing. Each panel shows the results for the indicated timepoint (T0 in panel B, T1 in panel C, T2 in panel D, T3 in panel E, and T4 in panel F). Each circle represents an ARG, its color shows the arm in which it was more prevalent, and its size is proportional to the odds ratio (OR) after logarithmic transformation. In each panel, ARGs with levels below the quantification threshold in > 25% of the samples were omitted as long as their frequency of absence between the two groups was not statistically different (p < 0.05). Dashed horizontal lines indicate q = 0.10. Datapoints above this threshold are statistically significant.

**Figure 3.**
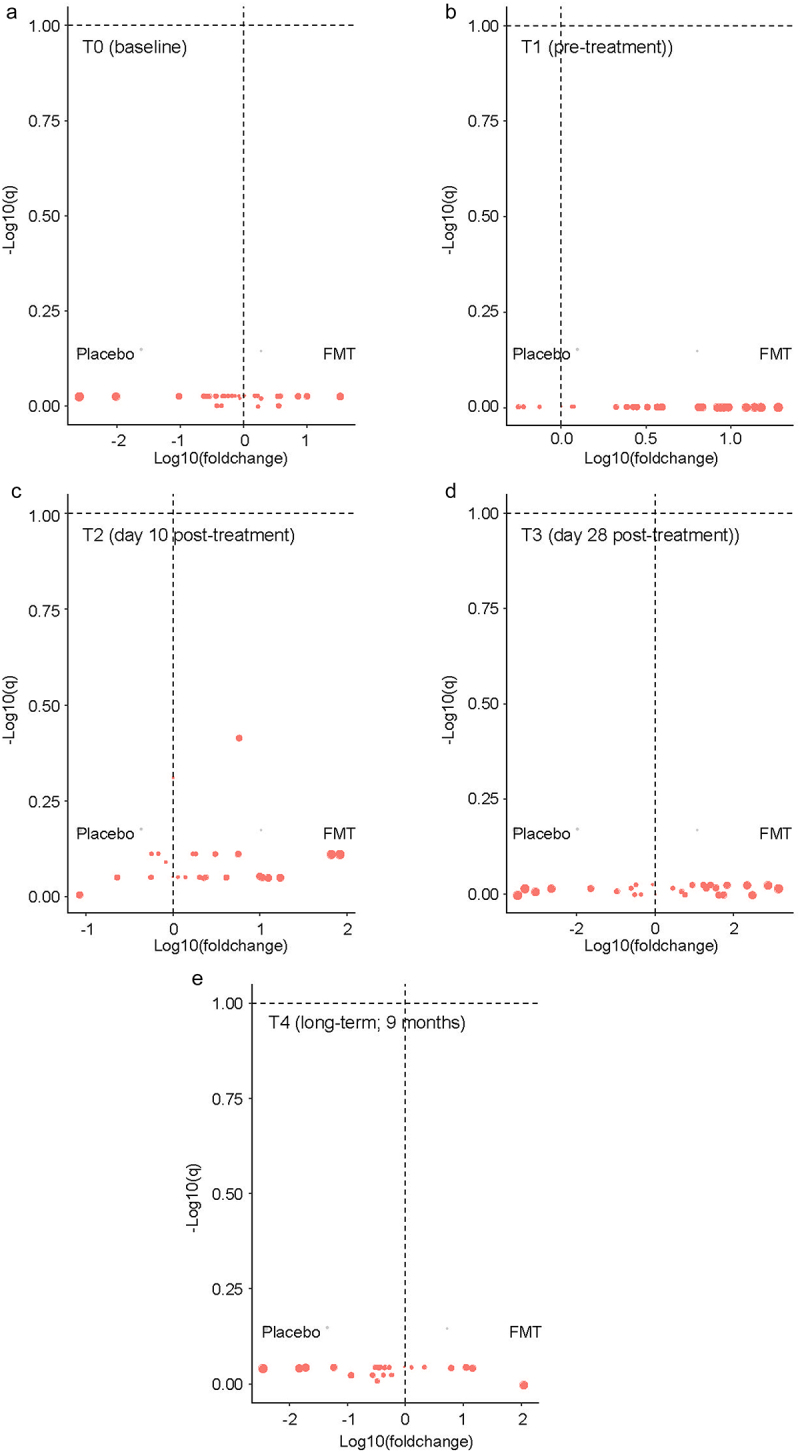
Cross-sectional quantitative comparison between the two arms.

**Figure 4.**
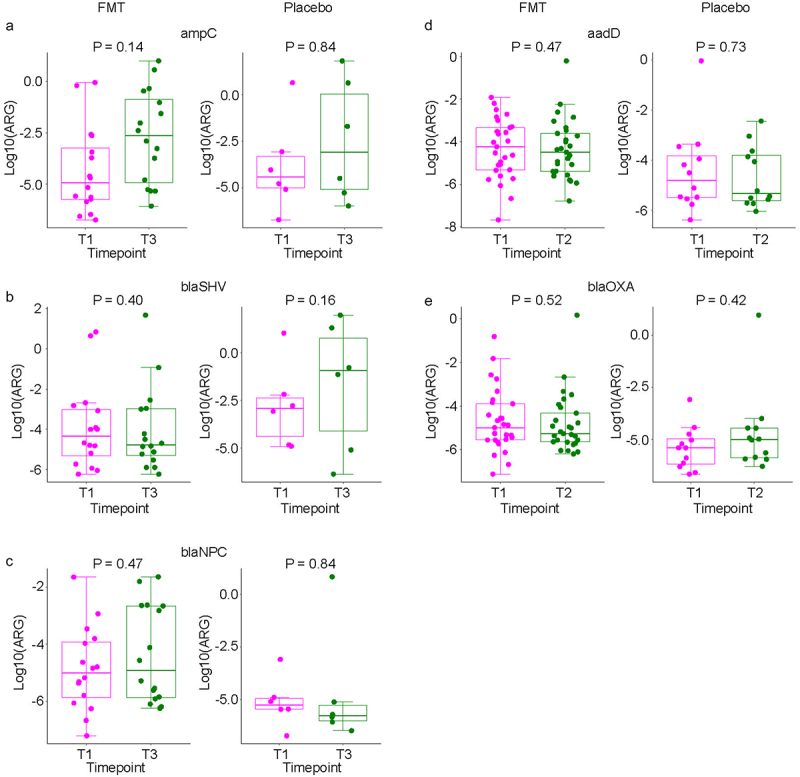
Longitudinal analysis of ARG quantitative levels comparing T1 (pre-dose 1) to subsequent timepoints.

In cross-sectional quantitative analysis at each timepoint, the level of none of the ARGs was significantly different between the two arms at any of the timepoints (Figure 3). In longitudinal analysis in mixed-effects regression comparing each post-treatment timepoint (T2, T3, and T4) to the pretreatment timepoint (T1) while accounting for same-patient repeated measurements (Table 2), the only ARG with a significant change was *nikA*, increasing from T1 to T2 (regression coefficient 0.37, 95%CI 0.04 to 0.71, *p* = 0.03) with no difference between the two arms. However, an interaction effect by treatment arm appeared to be present for *ampC* (T3), *blaSHV* (T3), *blaNPC* (T3), *aadD* (T2), and *blaOXA* (T2). Examination of the dynamics of these ARGs in the two arms separately did not support an interaction effect (Figure 4).

Each panel shows the results for the indicated timepoint (T0 in panel a, T1 in panel b, T2 in panel c, T3 in panel d, and T4 in panel e). Each circle represents an ARG. Data points to the right of the vertical dashed lines indicate ARGs with higher quantity in the FMT arm; those to the left have a higher quantity in the placebo arm. *p* values were estimated from a Wilcoxon test followed by false discovery correction to derive q values (y axis). The x-axis shows fold change in the quantity of each ARG after logarithmic transformation; the size of each circle is proportional to this value. Dashed horizontal lines indicate q = 0.10. Datapoints above this threshold (shown in magenta) are statistically significant. The size of each circle is proportional to the log (foldchange), the arm with a higher level relative to the arm with the lower level.

Table 4 shows the results of mixed-effects regression for all ARGs. The model suggested an interaction between treatment arm and timepoint, meaning ARG dynamics may differ between the two arms. Those 5 ARGs are examined individually in this figure. Each panel (a-e) shows the results for one ARG. In each panel, the plot on the left shows the results for the FMT arm, and the right plot shows the results for the placebo arm. Panels a, b, and c compare T3 (day 28 after treatment) to T0 (pre-treatment). Panels d and e compare T2 (day 10 after treatment) to T0. Each comparison is from a mixed-effects model for ARG level without interaction terms, where patient number is the random effect and timepoint is the fixed effect. *p* values for the regression coefficients representing timepoint (T2 or T3 vs. T1) are shown. The horizontal line within each boxplot shows the median.

**Table 2.** Mixed-effects modeling of quantitative ARG levels comparing T1 to subsequent timepoints.

ARG	Placebo vs. FMT (95%CI)	P	T2 vs. T1 (95%CI)	P	T3 vs. T1 (95%CI)	P	T4 vs. T1 (95%CI)	P
chrA	4.0 (−6.5 to 14.6)	0.45	0.3 (−1.5 to 2.1)	0.75	1.9 (−0.3 to 4.1)	0.09	0.8 (−1.5 to 3.1)	0.48
mefE	−0.02 (−0.06 to 0.02)	0.35	−0.01 (−0.06 to 0.03)	0.56	−0.02 (−0.08 to 0.03)	0.41	−0.01 (−0.06 to 0.04)	0.65
intl3	−11 (−37 to 16)	0.42	4 (−25 to 33)	0.78	−0.8 (−35.7 to 34.2)	0.96	21 (−10 to 52)	0.19
tetX	−19	0.46	8	0.79	−1	0.98	45	0.16
qnrB	−15 (−62 to 32)	0.52	0.6 (−48.9 to 50.1)	0.98	6 (−54 to 66)	0.85	47 (−6 to 100)	0.08
sul1	−0.9 (−2.7 to 0.8)	0.30	0.5 (−1.3 to 2.3)	0.58	−0.007 (−2.14 to 2.12)	0.99	1.5 (−0.4 to 3.4)	0.13
aacA	−1938 (−5922 to 2047)	0.34	−3846 (−8132 to 441)	0.08	−4499 (−9649 to 652)	0.09	−3979 (−8554 to 596)	0.09
floR	−1 (−6 to 3)	0.57	3 (−2 to 9)	0.21	−2 (−6 to 6)	0.99	−10 (−6 to 5)	0.99
blaNDM-1	−15 (−51 to 22)	0.42	26 (−17 to 68)	0.23	20 (−30 to 70)	0.43	0.9 (−43.6 to 45.4)	0.97
rcnA	−0.49 (−1.73 to 0.74)	0.43	0.03 (−1.33 to 1.38)	0.97	−0.01 (−1.64 to 1.62)	0.99	1.2 (−0.3 to 2.6)	0.11
blaKPC	0.008 (−0.01 to 0.02)	0.22	<-0.001 (−0.005 to 0.005)	0.99	0.004 (−0.002 to 0.01)	0.16	0.001 (−0.004 to 0.007)	0.69
nikA	−0.09 (−0.37 to 0.20)	0.54	0.37 (0.04 to 0.71)	0.03*	0.07 (−0.32 to 0.47)	0.71	0.08 (−0.27 to 0.43)	0.66
CTXM-32	−2 (−8 to 3)	0.41	0.03 (−6.2 to 6.3)	0.99	−0.1 (−7.6 to 7.4)	0.97	6.1 (−0.6 to 12.7)	0.07
tetM	−2 (−7 to 3)	0.44	0.6 (−0.8 to 2.0)	0.40	0.6 (−1.1 to 2.4)	0.49	0.9 (−0.9 to 2.7)	0.32
tetA	−35 (−102 to 32)	0.30	−75 (−152 to 3)	0.06	−75 (−167 to 18)	0.11	−72 (−154 to 10)	0.08
aadA5	−0.20	0.55	−0.003	0.99	0.01	0.97	0.61	0.14
sul3	−18 (−76 to 40)	0.53	−5 (−67 to 58)	0.89	−1 (−77 to 74)	0.97	48 (−19 to 115)	0.16
tetL	−0.19 (−0.65 to 0.27)	0.41	−0.05 (−0.50 to 0.40)	0.83	−0.01 (−0.56 to 0.53)	0.96	0.30 (−0.19 to 0.79)	0.22
strB	−3 (−10 to 4)	0.37	−6 (−14 to 2)	0.14	−6 (−16 to 3)	0.18	−5 (−13 to 3)	0.22
tetS	−5 (−17 to 7)	0.41	−0.04 (−12.2 to 12.1)	0.99	−0.1 (−14.7 to 14.5)	0.99	12 (−1 to 25)	0.08
ereB	−4.3	0.37	2.0	0.72	−4.0	0.55	−3.9	0.52
tetW	−91	0.09	−57	0.37	22	0.77	−0.48	0.47
aadD	n.a.	n.a.	n.a.	n.a.				
− FMT					−0.019	0.36	−0.019	0.29
− Placebo					−0.06 (−0.18 to −0.06)	0.32	−0.06 (−0.17 to 0.05)	0.26
ampC	n.a.	n.a.			n.a.	n.a.		
− FMT			1.9	0.23			1.1	0.50
− Placebo			−0.2 (−8.3 to 7.9)	0.95			3 (−5 to 12)	0.41
blaOXA	n.a.	n.a.	n.a.	n.a.				
− FMT					−0.04	0.80	−0.04	0.78
− Placebo					−0.02 (−1.24 to 1.19)	0.97	−0.02 (−1.10 to 1.06)	0.97
blaSHV	n.a.	n.a.			n.a.	n.a.		
− FMT			1.0 (−3.4 to 5.4)	0.65			4 (−1 to 9)	0.12
− Placebo			−0.5 (−13.5 to 12.5)	0.93			7 (−6 to 21)	0.28
blaNPC	n.a.	n.a.			n.a.	n.a.		
− FMT			0.01 (−0.19 to 0.21)	0.92			0.003 (−0.21 to 0.22)	0.98
− Placebo			0.04 (−0.69 to 0.76)	0.92			0.01 (−0.75 to 0.77)	0.98

The interaction between T2 and treatment arm was significant for aadD (p = 0.04) and blaOXA (p = 0.03). The interaction between T3 and treatment arm was significant for ampC (p = 0.04), blaSHV (p = 0.05), blaNPC (p = 0.001). For these 5 ARGs, comparison between the two arms at the timepoint with interaction with treatment arm was done separately in Figure 4. In all other cases, the interaction between timepoints and treatment arm was not significant, thus 95% confidence intervals (95%CI) and *p* values were estimated from a model without interaction terms. In some cases, the confidence interval could not be calculated. *p < 0.05.

## Discussion

This study is unique from at least two aspects. First, with baseline, pre-treatment, and 3 post-treatment samples spanning a period of 9 months, we were able to evaluate both short- and long-term dynamics of ARGs. Second, spontaneous microbiome recovery after the initial antibiotic injury likely influences ARG dynamics. Without a placebo group to represent this trajectory, the added or unique effect of FMT on ARGs and their dynamics is difficult to ascertain. Correlative analysis of longitudinal samples from a large, randomized placebo-controlled FMT trial allowed us to address this knowledge gap. As pre-dose 1 antibiotic exposures were comparable between the two arms and we had no samples in our final list with preceding antibiotic exposure after dose 1, our findings are not biased by antibiotic exposures. Randomization also reduced the risk of bias due to heterogeneity resulting from differences in diet and chemotherapy regimens, both of which can modulate the gut microbiota.^17,18^ Using prevalence and quantitative analysis, combined with cross-sectional and longitudinal approaches, our findings overall reveal the following dynamics. In the placebo arm, representing natural recovery of the microbiota after antibiotic injury, there was an initial decline in the prevalence of ARGs during antibiotic exposure (T0 to T1). ARG prevalence remained lower than baseline for at least a month after antibiotic discontinuation (T3). In the following months, the prevalence of ARGs increased and reached baseline levels at T4 (9 months post- treatment). In the FMT arm, the same pattern was seen before the study intervention (T0 to T1), supporting the internal validity of our analysis and success of randomization from a microbiome standpoint. In the first month after FMT (T1 to T3), ARG prevalence increased. This transient phase was then reversed, leading to communities with significantly fewer types of ARGs compared to the placebo arm.

These findings suggest a model where transmission of commensal bacteria harboring ARGs from the FMT product results in short-term increased the diversity of the gut resistome. This reflects engraftment of healthy donor-derived resistomes. Quantitative changes in ARG levels were not statistically significant, suggesting low-level, albeit broad ARG transmission. In the weeks to months to come, and as colonization resistance is established due to the formation of stable microbial communities, ARG diversity declines to levels lower than in the placebo arm.

The gut microbiota in healthy adults is a reservoir for multiple ARGs.^19–22^ Studies in hunter-gatherer populations have shown that a major part of the gut resistome has its origin in soil and water habitats, and some in food. As these populations had little exposure to antibiotics (or medications in general) in their lifestyle or food, their ubiquitous ARGs such as tetracycline and several beta-lactam resistance genes are environmentally derived.^23^ Only a small fraction of ARGs pose a threat to human health.^24^ A recent omics-based microbial ecology approach using four criteria (enrichment in human-associated environments, gene mobility, and presence/absence in ESKAPE Pathogens [host pathogenicity]) classified ARGs into 4 ranks based on their perceived risk to humans, with Rank I indicating highest risk ARGs and Rank IV representing lowest risk ARGs.^14^ This approach identified only 3.6% of ARGs as current and future threats to human health. These ARGs are commonly harbored by bacteria considered to be urgent or serious threats to human health (https://www.cdc.gov/drugresistance/biggest-threats.html); examples include carbapenem-resistant *Acinetobacter*, carbapenem-resistant or ESBL-producing *Enterobacterales*, vancomycin-resistant *Enterococci*, multidrug-resistant *Pseudomonas aeruginosa*, and methicillin-resistant *Staphylococcus aureus*. *vanA* and *sul1* were classified as Rank IV ARGs due to their lack of significant enrichment in human-associated environments. Our findings regarding specific ARGs support the proposed model. The six ARGs that had a higher prevalence in the month after FMT than placebo were *sul1* (sulfonamide resistance), *sul3* (sulfonamide resistance), ***floR*** (chloramphenicol resistance), *aadD* (aminoglycoside resistance), ***ermB*** (macrolide resistance), and *mefE* (macrolide resistance). *sul1 and sul3* are associated with some of the broadest taxonomic ranges both in the human gut microbiota and environment.^25^ Their wide-spread presence in the healthy gut microbiota^26^ strongly suggest their increase in prevalence after FMT to be due to transmission via FMT, facilitated by the expected poor colonization resistance of the microbiota after extensive antibiotic exposures typically experienced by our patient population. ***ermB*** was recently found in human pathogens, suggesting that it is at elevated risk of transferring into pathogens, thus it was classified as a Rank I ARG.^14^ Importantly, the initial increase in the prevalence of these ARGs after FMT did not last long, as by T4, none were more prevalent than in the placebo arm. In addition, quantitative assessment suggested that the emergence of these ARGs in some early post-FMT samples was at low levels. Reassuringly, clinically important ARGs conferring vancomycin, fluoroquinolone, or carbapenem resistance did not become more prevalent after FMT.

The eventual decline in ARG prevalence to levels lower than the placebo arm may be due to the disappearance of some of the transmitted ARGs or resistance to new ARGs. The marked rise in the total number of ARGs between T3 and T4 in the placebo arm compared to a stable plateau in the FMT arm supports the latter scenario. Rewiring of the microbiota networks after FMT, as we reported recently,^27^ likely results in establishment of stable communities with substantial colonization resistance. Restoration of these properties protects the microbiota against invasion by MDROs and expansion of ARGs within the community. However, we cannot eliminate the possibility of donor-derived resistomes shrinking over time. The multitude of microbiota-relevant events in the long period of follow-up (9 months) limited our ability to distinguish between these two explanations.

This study has some limitations. First, although our ARG panel was larger than many previous reports, the gut resistome is much larger. Second, data in this work came from the analysis of fecal material. Microbiota within fecal material is not representative of all bacterial populations in the gut. Mucosal-adherent bacteria may be more relevant to host physiology and many of them are likely underrepresented in stool.^28^ Third, we did not have sufficient material from donors to evaluate the same ARG in donor material. Therefore, our inference of ARG transmission via FMT could not be proven by direct empirical evidence. Fourth, the FMT dose might have been insufficient for a significant clinical effect. Although this dose has been sufficient for the treatment of patients with rCDI, the unique clinical context in AML and transplant patients (e.g., chemotherapy, often severe gut barrier damage, and great antibiotic pressure) makes the host less receptive to new microbiota, thus requiring a higher dose. A higher microbiota dose may lead to better engraftment, with a greater potential to modulate clinical outcomes.

In conclusion, our findings suggest that FMT led to a low-grade transmission of ARGs largely ubiquitous to the commensal bacteria, reflecting an expected engraftment of healthy donor-derived resistomes. This initial phase was followed by long-term acquisition of resistance to clinically relevant ARGs associated with MDROs, consistent with the FMT experience in patients with CDI.^29^ While clinical response in patients with recurrent CDI receiving FMT has been associated with a reduction in the number of ARGs,^30^ the clinical significance of long-term ARG decolonization by FMT in patients with AML and alloHCT recipients is unknown. In our randomized trial, FMT was associated with a numerical, albeit not statistically significant, decrease in infection rates in the first 4 months. Whether this trend becomes significant over time remains to be studied in future research.

## Ethics approval

The trial protocol (ClinicalTrials.gov identifier: NCT03678493) was approved by the University of Minnesota Institutional Review Board and complied with local regulations and the Declaration of Helsinki. All participants provided written informed consent.

## Availability of data and materials

All raw data analyzed in this manuscript including gene copy numbers and related clinical metadata are provided in supplementary data S1.

## Supplementary Material

Supplemental Material

Supplementary Data S1.xlsx
