# Supplementary material for: Long- and short-term effects of fecal microbiota transplantation on antibiotic resistance genes: results from a randomized placebo-controlled trial
Source: Gut Microbes. 2024 Mar 13;16(1):2327442. doi: 10.1080/19490976.2024.2327442 (PMC10939144; doi:10.1080/19490976.2024.2327442)
Supplement: Supplemental Material [file KGMI_A_2327442_SM1195.docx]

1.00

0.75

0.50

Rank I ARGs (%)

0.25

0.00

# P = 0.98


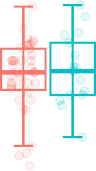


P = 0.97


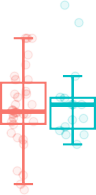


# P = 0.71


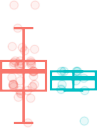


# P = 0.13


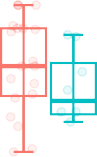


# P = 0.39


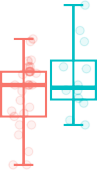


Arm

# FMT

- Placebo

# T0 T1 T2 T3 T4

Timepoint

Supplementary Fig. S1: Comparison between the two arms at each timepoint for the proportion of rank I ARGs

The proportion of rank I ARGs relative to all ARGs present at each timepoint is compared between FMT and placebo arms using a Wilcoxon test. The horizontal line in each boxplot shows the median.
